# Innate immunity pathways activate cell proliferation after penetrating traumatic brain injury in adult *Drosophila*

**DOI:** 10.1080/19336934.2025.2586357

**Published:** 2025-11-14

**Authors:** Shawn Ahern-Djamali, Khailee Marischuk, Kassi L. Crocker, Isabella Peetz, Eli Scott, Grace Boekhoff-Falk

**Affiliations:** a Department of Cell and Regenerative Biology, University of Wisconsin-Madison, School of Medicine and Public Health, Madison, WI, USA; b Genetics Graduate Training Program, University of Wisconsin-Madison, School of Medicine and Public Health, Madison, WI, USA; c Science and Medicine Graduate Research Scholars Program, University of Wisconsin-Madison, School of Medicine and Public Health, Madison, WI, USA

**Keywords:** Drosophila, adult neurogenesis, innate immunity, brain, mushroom body

## Abstract

We are utilizing an adult penetrating traumatic brain injury (PTBI) model in Drosophila to investigate regenerative mechanisms after damage to the central brain. Here, we focus on cell proliferation as an early event in the regenerative process. To identify pathways that could trigger cell proliferation following PTBI, we utilized bulk RNA-Seq. We find that transcript levels for components of both Toll and Immune Deficiency (Imd) innate immunity pathways are rapidly and highly upregulated post-PTBI. We then tested mutants for the NF-κB transcription factors of the Toll and Imd pathways, Dorsal-related immunity factor (Dif) and Relish (Rel), respectively. We find that loss of either Dif or Rel results in loss of cell proliferation after injury and identify tissue-specific requirements for Dif and Rel. In addition, while the canonical downstream targets of *Drosophila* innate immune signalling, the antimicrobial peptides (AMPs), are upregulated following PTBI, their levels revert to near baseline within 24 hr. Taken together, these results indicate that the innate immunity pathways play an integral role in the regenerative response and that this response may not require the antimicrobial peptides. Innate immunity previously has been implicated as both a potentiator and an inhibitor of regenerative processes. Our work suggests that modulation of innate immunity may be essential to prevent adverse outcomes. Thus, this work is likely to inform future experiments to dissect regenerative mechanisms in higher organisms as well as in *Drosophila*.

## Introduction

The scarcity of neural stem cells in the adult brain represents an apparent barrier to therapeutic neural regeneration in humans. Treatments for neurodegenerative diseases and neural injuries thus have focused primarily on the transplantation of stem cells (reviewed in [1]). However, transplants are complex, costly, and may have undesirable side effects, including tumour formation (e.g [2]). For these reasons, therapies that activate endogenous regenerative mechanisms are an attractive alternative. Indeed, regeneration from resident cells is a growing area of research but remains technically challenging (reviewed in [3]). *Drosophila* are amenable to genetic techniques not easily utilized in higher organisms, and the adult *Drosophila* central brain, like the adult mammalian brain, has few neural stem cells. We therefore developed an adult *Drosophila* model in which to investigate brain regeneration.

Following a penetrating traumatic brain injury (PTBI), we found that cells in the adult *Drosophila* central brain are stimulated to proliferate [4,5]. PTBI of the adult Drosophila optic lobes also results in cell proliferation and the formation of new neurons [6], while PTBI of the adult central brain gives rise to both new glia and new neurons [5]. The mechanism that activates cell proliferation post-PTBI is unknown; however, our preliminary studies suggested that innate immune signalling pathways are involved. The *Drosophila* innate immune system comprises both cellular and humoral components and utilizes multiple signalling pathways, including the Janus kinase/signal transducers and activators of transcription (JAK/STAT), Jun N-terminal kinase (JNK), Toll, and Immune deficiency (Imd) pathways (reviewed in [7–9]). In this work, we focus on the roles of the Toll and Imd pathways in the post-PTBI proliferative response.

The Toll pathway can be stimulated by microbial peptidoglycans or damage-associated molecular patterns (DAMPs) via the activation of an extracellular ligand, Spätzle (Spz). A signalling cascade leading to cleavage of Spz is activated by the Peptidoglycan recognition protein-SA (PGRP-SA) in conjunction with other molecules and involves multiple proteases, including Spätzle-Processing Enzyme (SPE), to activate immune signalling (reviewed in [7]). Cleaved Spz is recognized by the Toll receptors at the cell membrane, triggering a pathway that results in the degradation of Cactus, a negative regulator of two nuclear factor-kappa B (NF-κB) transcription factors, Dorsal and Dorsal-related immunity factor (Dif). Translocation of Dif to the nucleus results in the transcriptional upregulation of canonical immune signalling target genes, which encode antimicrobial peptides (AMPs) [10]. AMPs can kill microbes, thus removing pathogenic threats. Both Dorsal and Dif regulate immune signalling in embryos and larvae, with Dif proposed to be more important in adults for immune function [11]. Of potential relevance to this study, *Dif* was recently shown to play a role in developmental neurogenesis with loss of function mutations in *Dif* resulting in smaller numbers of neurons in the adult brain, decreased locomotor ability and a shorter lifespan [12].

The Imd pathway is also activated by microbial peptidoglycans, with relevant receptors such as PGRP-LE either freely distributed in the haemolymph or alternatively like PGRP-LC located in the cellular membrane (reviewed in [13–16]). In this case, microbial peptidoglycan binding causes a complex of Imd, Death related ced-3 (Dredd), and Fas-associated death domain (Fadd) to be recruited. This complex activates downstream kinases, leading to the phosphorylation and cleavage of a third NF-κB transcription factor, Relish (Rel). Once Rel is activated, it migrates to the nucleus to induce the expression of AMPs and other immunity genes. The Imd pathway has several potent negative regulators, including extracellular PGRP-SC2 and intracellular Pirk (reviewed in [13–16]).

Previous studies suggest that immune signalling in the brain can be detrimental to the tissue, and that immune system activation plays a role in many neurodegenerative diseases (reviewed in [17]). In Drosophila, overexpression of AMPs in glia results in neurodegeneration, and Rel activity underlies neurodegeneration in a fly model of ataxia-telangiectasia [18–21]. A closed head traumatic brain injury (TBI) in adult Drosophila results in sustained activation of innate immune signalling, also leading to neurodegeneration [22–29].

In contrast, innate immune signalling in embryos and larvae can stimulate wound closure and promote cell survival [30,31]. While a penetrating injury to the *Drosophila* optic lobe is sufficient to activate expression of AMPs and other genes associated with the immune response, it does not lead to neurodegeneration; this is in contrast to what is seen in the closed-head injury model [24]. Thus, although multiple studies implicate Toll and Imd activation in cell and/or organismal death following brain injury, a beneficial role for innate immune signalling in neurogenesis has not been previously demonstrated in *Drosophila*. Based on our initial studies correlating upregulation of innate immunity with cell proliferation following penetrating brain injury, we hypothesized that the Toll and Imd pathways can promote neurogenesis after brain injury. Consistent with this hypothesis, studies in mammals have demonstrated that immune activation and inflammation are capable of promoting either neurogenesis or neurodegeneration, depending on the microenvironment of the injury and the relative scale of the inflammatory response (reviewed in [32,33]).

Our work supports previous *Drosophila* brain injury studies in demonstrating that immune signalling pathways are upregulated after penetrating brain injury [24]. In addition, we find that the activation of Toll and Imd signalling through the NF-κB transcription factors, Dif and Rel, is required to trigger cell proliferation following PTBI. We further demonstrate that this signalling is required in specific cell types both within and surrounding the brain. Because the canonical targets of innate immune signalling, the AMPs, appear not to be required for cell proliferation post-PTBI, we conclude that there are likely to be other targets of Toll and Imd signalling that are essential for the response to PTBI. Based on the rapid upregulation of inhibitors of both Toll and Imd signalling and the transience of AMP upregulation post-PTBI, we propose that swift modulation of the immune response is essential to forestall secondary injury and subsequent neurodegeneration while promoting neuroregeneration. Taken together, this work illustrates that a major regulator of proliferation after brain injury is the innate immune system and that the precise spatial and temporal control of immunity cascades may trigger the initial steps required for neural regeneration after injury.

## Materials & methods

### Fly stocks and rearing

All flies were reared at 25°C on a standard cornmeal-sugar medium. The following stocks were obtained from the Bloomington Drosophila Stock Center: #458 (*p{GawB}elav[C155]*), #25374 (y[1] w[*]; P{Act5C-GAL4-w}E1/CyO), #7415 (*w*^*1118*^; P{w[+m*] = GAL4}repo/TM3, Sb^*1*^), #9458 (*w*^*1118*^; *Rel*^*E38*^ *e*^*s*^), #28943 (*y*^*1*^ *v*^*1*^; *P{y[+t7.7] v[+t1.8] = TRiP.HM05154}attP2*), #30141 (*w*^*1118*^; *P{w[+mC] = Hml-GAL4.Delta}3*), #30513 (*y*^*1*^ sc^*^ *v*^*1*^ *sev*^*21*^; *P{y[+t7.7] v[+t1.8] = TRiP.HM05257}attP2*), #34559 (*P{ry[+t7.2] = Dipt2.2-lacZ}1, P{w[+mC] = Drs-GFP.JM804}1, y*^*1*^ w^*^; *Dif*^*1*^ *cn*^*1*^ *bw*^*1*^), #36558 (*P{ry[+t7.2] = Dipt2.2-lacZ}1, P{w[+mC] = Drs-GFP.JM804}1, y*^*1*^ w^*^; *Dif*^*2*^ *cn*^*1*^ *bw*^*1*^), #51635 (*y*^*1*^ w^*^; P{w[+m*] = nSyb-GAL4S}3), #55707 (*P{ry[+t7.2] = Dipt2.2-lacZ}1, P{w[+mC] = Drs-GFP.JM804}1, y*^*1*^ w^*^), #55714 (*Rel*^*E20*^), and #58814 (*y*^*1*^ w^*^; *P{w[+mC] = yolk-GAL4}2*). Double mutant *Dif*^*1*^*/Dif*^*2*^; *Rel*^*E20*^*/Rel*^*E38*^ stocks were generated in our laboratory using the stocks listed above. The AMP deletion stocks were generously provided by the Lemaitre lab: Group A deletion strain (lacking *Defensin*), Group B deletion strain (lacking *Drosocin, Attacin A, Attacin B, Attacin C, Attacin D, Diptericin A*, and *Diptericin B*), Group C deletion strain (lacking *Metchnikowin* and *Drosomycin*), and Group ABC deletion strain. Full descriptions of these stocks are available in Hanson et al. [34]. We note that Group A also includes the Cecropins, which were not deleted in either the Group A or Group ABC deletion stocks used here [34].

### Penetrating traumatic brain injury

To induce PTBI, we used thin metal needles (~12.5 μm diameter tip, 100 μm rod; Fine Science Tools) sterilized in 70% ethanol to penetrate the head capsule of CO_2_-anesthetized adult flies [4]. Injured flies were transferred back to our standard sugar food for recovery and ageing. The same injury method was applied to flies for 5-ethynyl-2’-deoxyuridine (EdU) labelling, except flies were fed 50 mM EdU in 10% sucrose solution on a size three Whatman filter for 6 hr before injury, then allowed to recover on the same solution for 24 hr. For immunohistochemistry experiments, each fly was unilaterally injured in the right hemisphere of the central brain. For molecular experiments, each fly was injured bilaterally.

### Bulk RNA-Seq

Bulk RNA-Seq samples were isolated from the heads of males 4 hr after PTBI to both hemispheres of the central brain and from the heads of age- and sex-matched uninjured controls. RNA-seq workflow integrated service was provided by ProteinCT Biotechnologies LLC (Madison, WI). Libraries were prepared using the TruSeq strand-specific mRNA sample preparation system (Illumina). The final library was generated by further purification and amplification with PCR, and quality checked using a Bioanalyzer 2100. The libraries were then sequenced (single end 50 bp reads) using the Illumina HiSeq2500, with six samples (three control and three injured) per lane, for a total of over 20 million reads per sample. Next, the fast QC programme was used to verify the raw data quality of the Illumina reads. The *Drosophila melanogaster* genome and gene annotations were downloaded from FlyBase and used for mapping. The raw sequence reads were mapped to the genome using Subjunc aligner from Subread, with the majority of the reads (over 98% for all samples) aligned to the genome. The alignment bam files were compared against the gene annotation GFF file, and raw counts for each gene were normalized using the voom method from the R Limma package. Once normalized, they were then used for differential expression analysis. Hierarchical clustering was used to indicate sample and gene relationships. In the overall heatmap, each column is a sample, and each row represents the scaled expression values for one gene (blue is low, red is high). In this clustering, there were 367 genes with differential expression of 2-fold or greater between the two groups. Two hundred and fifty-nine of those were upregulated, and 108 were downregulated with an adjusted *p* value < 0.05.

### Immunohistochemistry

Brains were dissected in PBS and fixed in a 3.7% formaldehyde in a PEM (100 mM piperazine-N,N’-bis(2-ethanesulfonic acid) [PIPES], 1 mM EGTA, 1 mM MgSO_4_) solution for 20 min at 25°C. Fixed brain samples were washed in PT (PBS [phosphate-buffered saline] and 1% Triton X-100), blocked with 2% BSA in PT solution (PBT), and then incubated with primary antibodies overnight at 4°C in PBT. Following primary incubation, the samples were washed with PT and then incubated overnight in a secondary antibody at 4°C. The next day, samples were washed in PT, stained with DAPI (1:10,000, ThermoFisher) for 8 min, and mounted in Vectashield anti-fade mountant (Vector Labs). The primary antibodies used in this study are rabbit anti-PH3 (1:500, Santa Cruz Biotechnology, Inc). Secondary antibodies used are anti-rabbit 568 (1:400, ThermoFisher). For EdU labelling, brains were dissected, processed, and antibody stained as described above using buffers without azide prior to the Click-IT® reaction. EdU detection was performed by following the manufacturer’s protocol (InVitrogen). All slides were imaged using a Nikon A1RS system and analysed using the Nikon NIS Elements software. Cell counting was done both manually and using the Nikon NIS-Elements software to detect regions of interest (ROIs) with a threshold of over 1000 and an area of at least 10 μm.

### Quantitative real-time PCR

Transcript levels of target genes were measured by quantitative real-time PCR (qRT-PCR) using methods described in [35]. For each biological replicate, 20 males were subjected to PTBI to both hemispheres of the central brain within 6 hr of the eclosion and the heads were harvested 4 hr later. RNA was isolated using the RNeasy Plus Mini Kit (Qiagen) following homogenization of the tissue in Trizol using a motorized pestle (UW Biotech Center). The integrity and concentration of the RNA were determined spectrophotometrically using a NanoDrop and associated software (NanoDrop Technologies). First-strand cDNA was synthesized from 400 ng of total RNA using an oligo(dT) primer and the SuperScript III First-Strand Synthesis System (Invitrogen) according to the manufacturer’s instructions. qPCR was performed on a BioRad CFX Connect Real-Time System (BioRad). In all cases, technical triplicates of each sample were run simultaneously from three independent biological replicates for each target gene, and *Rp49* was used as the reference gene. Relative expression changes were calculated using the comparative CT method (2^(-ΔΔCT). The following primers were used for qRT-PCR: *Rel* Forward: 5’-TCCTTAATGGAGTGCCAACC-3’ & Reverse: 5’-TGCCATGTGGAGTGATTAT-3’ (Fly Primer Bank); *spz* Forward: 5’-GCGATTCCTTTGCAGGAGC-3’ & Reverse: 5’-AATTAACTGCCAGGTGTCGTC-3’ (Fly Primer Bank); *pirk* Forward: 5’-TACCGCATTGAGAACAACACG-3’ & Reverse: 5’-GGATCTTGAGTCTGGTGCTATTG-3’ (Fly Primer Bank); *PGRP-SC-2* Forward: 5’-CCACAGCGCTGGAAACTACT-3’- & Reverse: 5’-CCGCCGATCAGGAAGTTGTA-3’ (Fly Primer Bank); *PGRP-LC* Forward: 5’-AGGCCGTCACAGTTACAGTG-3“ & Reverse: 5”-GTGGTGGCCAGTACGATACC-3“; *Spn88Ea* Forward: 5”-CCGTACAGCACGTATCATGC-3’ & Reverse: 5’-GAGTCGGCCCAATCCAGAT-3 (Fly Primer Bank); *DptA* Forward: 5’-GCTGCGCAATCGCTTCTAC-3’ & Reverse: 5’-TGGTGGAGTGGGCTTCATG-3’ [36]; *CecB* Forward: 5’- TTGTGGCACTCATCCTGG-3’ & Reverse: 5’-TCCGAGGACCTGGATTGA-3’ [37]; *Dro* Forward: 5’-GCACAATGAAGTTCACCATCGT-3’ & Reverse: 5’-CCACACCCATGGCAAAAAC-3’ [38], and *Rp49* Forward: 5’-CCAGTCGGATCGATATGCTAA-3’ & Reverse: 5’-ACGTTGTGCACCAGGAACTT-3’ [39].

## Results

### Innate immunity genes are upregulated after PTBI

As reported in Crocker *et al*. [5] we have developed a novel method to study neurogenesis and gliogenesis in the adult Drosophila brain. We use a fine sterile needle to inflict penetrating traumatic brain injury (PTBI) on the mushroom body (MB) region of the young adult central brain [5]. After injury, we see a regenerative process that begins with an increase in the number of proliferating cells [5]. These new cells then differentiate into glia and neurons that appear to integrate into the brain circuitry and functionally repair the damage [5]. Notably, we do not see the previously reported neurodegenerative response following closed head traumatic brain injury (TBI) [22]. To elucidate how the regenerative process occurs, we focused on one of the first steps in the process: cell proliferation (Figure 1A). To identify genes that may activate proliferative mechanisms in the brain, we analysed changes in gene expression after PTBI via bulk RNA-Sequencing (RNA-Seq). We used RNA isolated from the heads of males 4 hr after bilateral injury to both mushroom body regions of the central brain and from age-matched, uninjured control heads of the same sex. The rationale for this early time point was to identify signalling pathways that might activate or otherwise contribute to cell proliferation post-PTBI. The GEO accession number for this dataset is: GSE306282 (https://www.ncbi.nlm.nih.gov/geo/). There were 367 genes with differential expression of 2-fold or greater between the injured and control groups. Of these, 259 were upregulated, and the remaining 108 were downregulated, with a false discovery rate (FDR) of <0.05. Gene Ontology (GO) terms for the upregulated genes in our injured samples were identified to determine what biological processes were affected upon injury. The single most highly enriched set of genes were defence and immune response, with 27% of identified GO Terms falling into this category. Components of both major innate immune pathways were upregulated 4 hr after injury along with the canonical downstream readouts of immune activation, the AMPs (Figure 1B,C).

**Figure 1.**
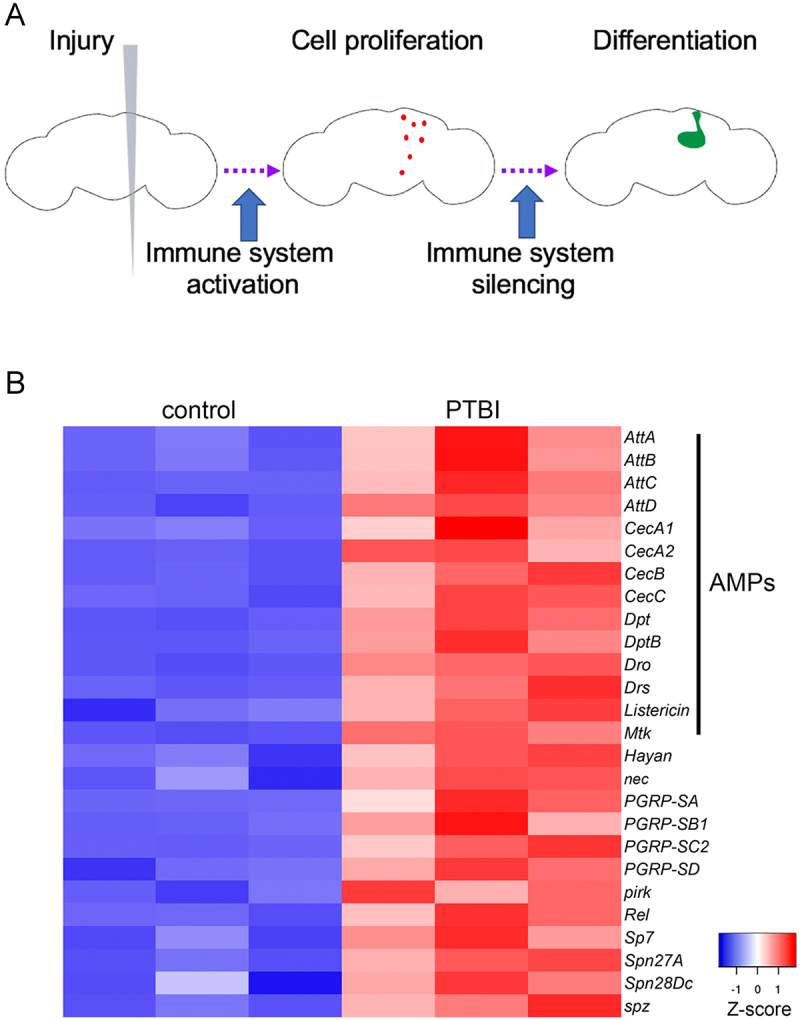
Penetrating traumatic brain injury (PTBI) induces cell proliferation and adult neurogenesis via activation of the immune system. A. The immune system is activated acutely and transiently after PTBI. We propose that this transient activation triggers proliferation of cells (red) particularly around the area of injury. The immune system is then silenced, as evidenced by the downregulation of innate immune pathway genes. The newly created cells can differentiate into new neurons and glia that go on to integrate and repair the damage to the mushroom body (green). B. Heat map showing differential expression of significantly (adjusted *p* value < 0.05) upregulated or downregulated genes in uninjured and 4-hr post-PTBI bulk RNA-Seq datasets. The three control replicas are on the left, and the three injured samples are on the right. Red indicates upregulation, while blue indicates downregulation. Multiple components of the Toll and Imd signalling pathways are upregulated. The antimicrobial peptides (amp) genes; canonical downstream targets of Toll and Imd signalling, also are upregulated by PTBI. The heat map plots Z-scores, which represent the number of standard deviations the expression for each sample is from the mean expression across all six samples. C. mRNA levels for multiple Toll and imd signalling pathway components are upregulated after PTBI. The mRNA levels of *Rel*, *spz*, *pirk*, *PGRP-SC2, PGRP-LC, and Spn88Ea* were assayed at 30 min, 2 hr, 4 hr, 8 hr, 24 hr, 3 d and 5 d post-PTBI. Transcript levels were all rapidly upregulated and returned to baseline by 24 hr after injury. The qRT-PCR results reflect triplicate biological samples, relative to the levels of *Rp49* mRNA in each sample. Relative expression changes for each mRNA were determined by comparison to its expression level in uninjured age and sex matched controls. Error bars reflect standard error of the mean (sem) and were calculated using prism software Tools.

**Figure 1.**
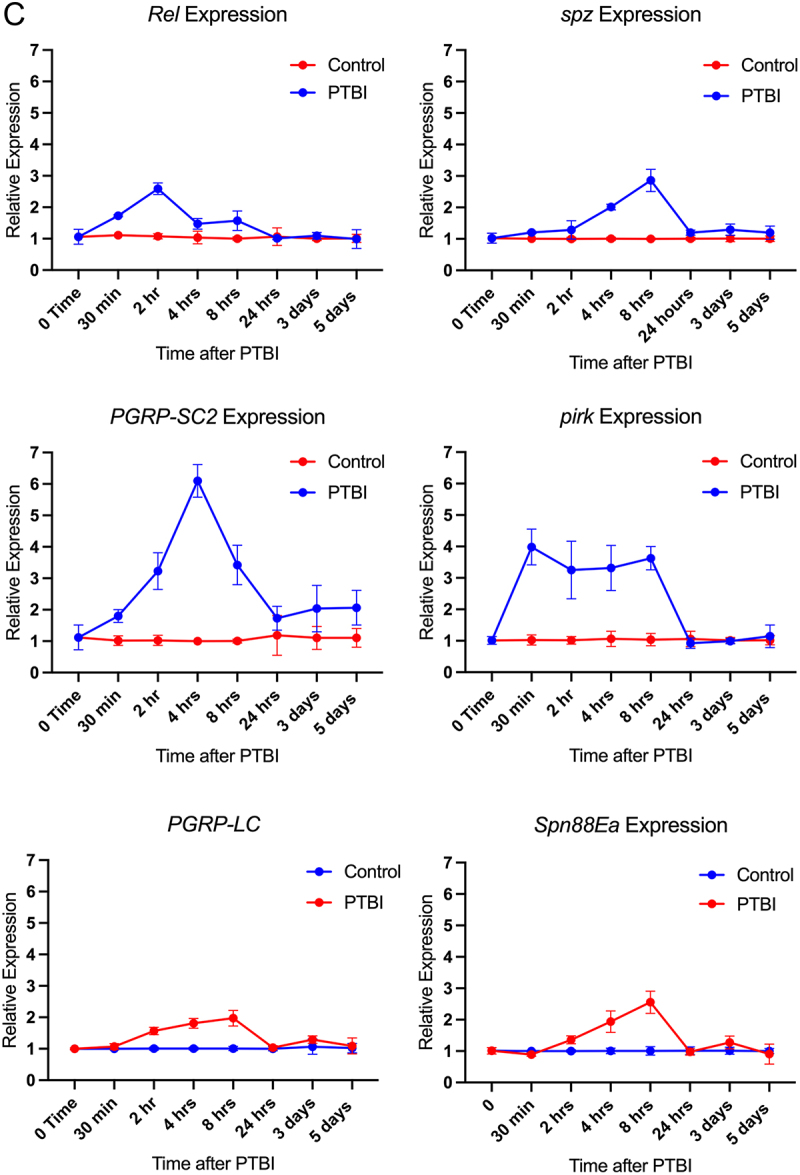
(Continued).

### Mutants in NF-κB transcription factor genes display proliferation defects after injury

**Figure 2.**
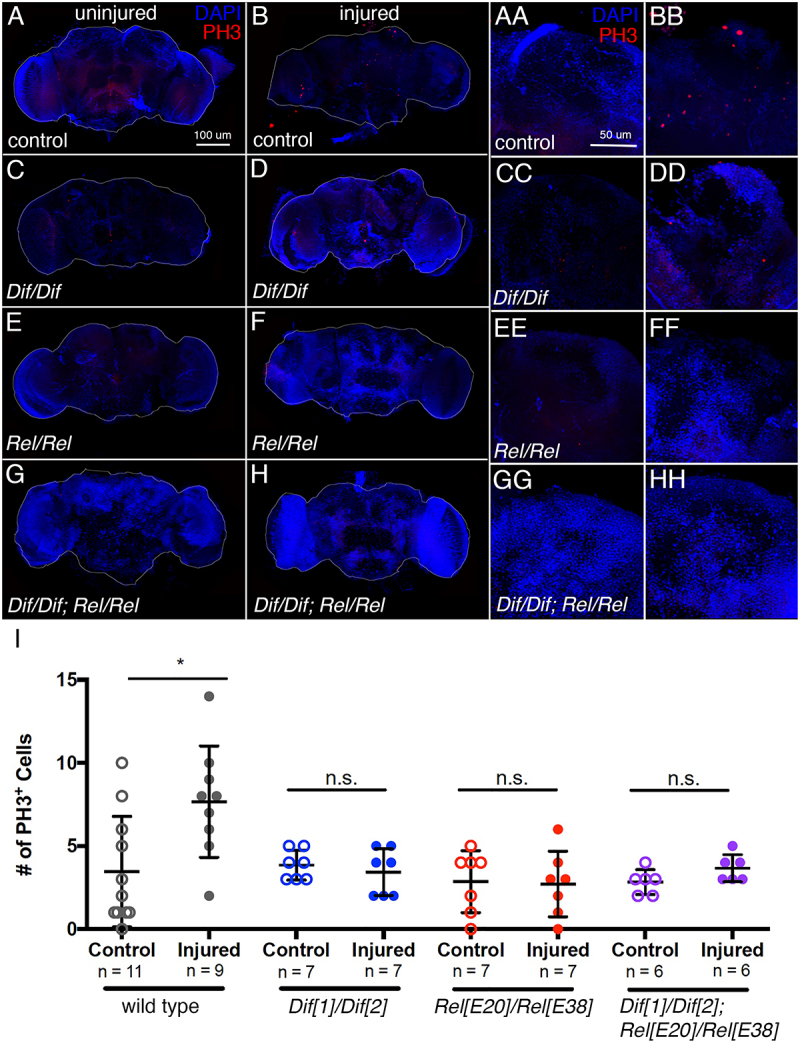
Dif and Rel mutants do not exhibit increased cell proliferation following PTBI. A-H. Cell proliferation assayed by anti-PH3 labelling (in red) 24 hr after injury reveals that while baseline cell proliferation (a) is not significantly reduced in uninjured Dif^1^/Dif^2^ (c), Rel^E20^/Rel^E38^ (e) and Dif^1^/Dif^2^; Rel^E20^/Rel^E38^ (g) double mutants, cell proliferation is not stimulated by PTBI in the mutants (D,F,H) as it is in control (b) animals. The right mushroom bodies of each of these brains are shown in higher magnification in AA-HH. I. Quantification of cell proliferation in Dif and Rel mutants reveals no statistically significant increase after injury. Wild-type control brains had an average of 3.5 PH3+ cells per brain, and wild-type injured samples had 7.7 (p-value = 0.01). Dif^1^/Dif^2^ mutant control brains had 3.9 PH3+ cells per brain, and injured samples had 3.4 PH3+ cells (p-value = 0.51). Rel^E20^/Rel^E38^ mutant control brains had 2.9 PH3+ cells on average, and injured brains had 2.7 PH3-positive cells (p-value = 0.89). The Dif^1^/Dif^2^; Rel^E20^/Rel^E38^ double mutant control samples had 2.8 PH3-positive cells per brain on average, and the injured samples had 3.7 PH3-positive cells (p-value = 0.10). Error bars reflect sd.

To test whether Toll or Imd signalling is necessary after brain injury for cell proliferation, we utilized mutants for the major effectors of signalling in each pathway. Because both pathways converge on NF-κB transcription factors, we used allelic combinations of mutants for *Dif* and *Rel* to block Toll and Imd signalling, respectively. We injured the mutant flies using our standard PTBI technique and allowed the flies to recover for 24 hr before assaying for cell proliferation. Two methods were used to detect cell proliferation. The first was an antibody to phospho-histone H3 (PH3), a histone variant present during the final stages of S phase through telophase of M phase [40]. In animals of the control genotype, we observed the stereotypical >2-fold increase in the number of proliferating cells in injured brains compared to uninjured controls (Figure 2A-BB,I). However, in *Dif* or *Rel* mutants, there was no significant increase in the number of PH3^+^ cells after injury (Figure 2C-FF,I). We also assayed flies mutant for both *Dif* and *Rel* and observed a similar lack of injury-stimulated proliferation in these samples (Figure 2G-HH,I). We note that baseline proliferation appeared to be unchanged in the mutants. Because PH3 is present for only part of the cell cycle, we were concerned that these assays would not capture the scope of proliferation. We therefore repeated the experiments using a second cell proliferation labelling technique, 5-ethynyl-2’-deoxyuridine (EdU), which is incorporated into newly synthesized DNA and permanently labels new cells and their daughters. We observed the same trend using this labelling technique, with fewer proliferating cells in the *Dif* and *Rel* mutants after injury as compared to the injured control brains (Fig. S1). We likewise saw no significant difference between control and injured *Dif; Rel* double mutants, indicating that the activation of proliferation in response to injury does not occur in the double mutant background (Fig. S1). We conclude that both Toll and Imd signalling are required for cell proliferation after PTBI. Because the numbers of proliferating cells detected were similar with anti-PH3 and EdU labelling, we used PH3 staining to detect proliferating cells for the remainder of the experiments described here.

### Immune system activation is transient after PTBI

**Figure 3.**
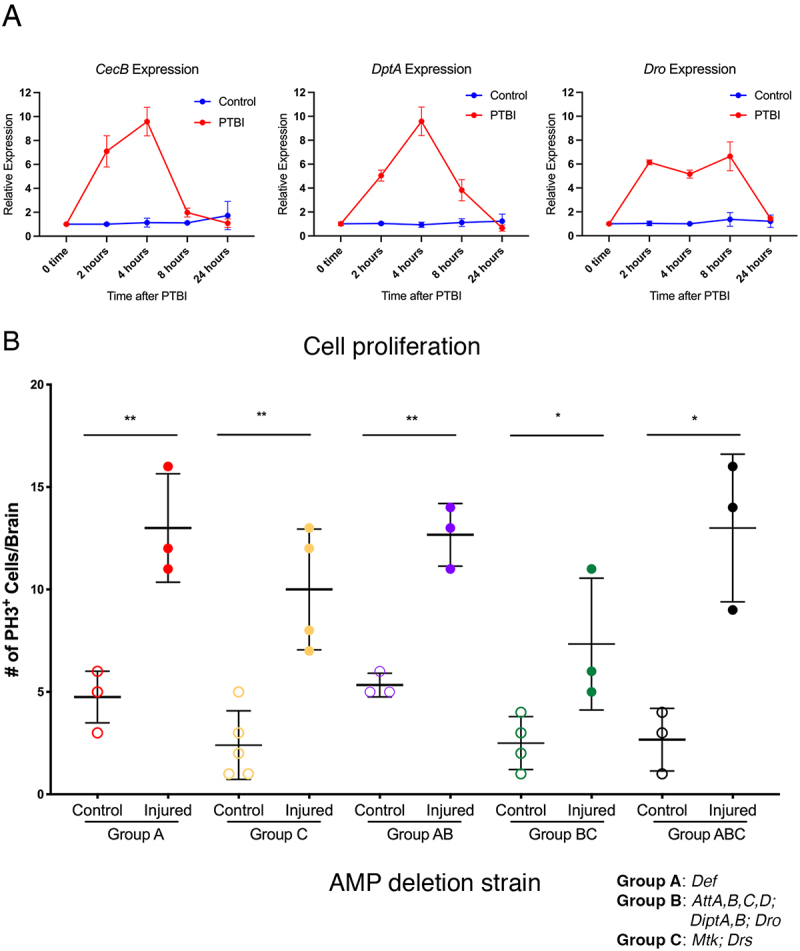
Antimicrobial peptides (AMPs) are not required for the proliferative response. A. Consistent with our bulk RNA-Seq analysis, the mRNA levels of three different AMPs assayed with qRT-PCR are upregulated after injury. For these experiments, we collected samples at 2, 4, 8 and 24 hr after injury. The levels of *CecB* and *DptA* mRNAs are increased 10-fold by 4 hr post-PTBI and return to near baseline by 8 hr. In contrast, the level of *Dro* mRNA increases ~6 fold by 2 hr and remains elevated at 8 hr with a drop to baseline by 24 hr post-PTBI. The qRT-PCR results reflect triplicate biological samples, represented relative to the levels of *Rp49*. Relative expression changes for each mRNA were determined by comparison to its expression level in uninjured age and sex matched controls. Error bars reflect sem and were calculated using prism software Tools. B. Fly stocks lacking groups of amp genes and combinations of these groups (see text for details) were assayed for cell proliferation 24 hr post-injury using PH3 staining. Loss of any single group of AMPs was not sufficient to reduce cell proliferation after injury. Nor did pairwise combinations of amp deletions (Group ab, Group bc) exhibit defects in cell proliferation post-PTBI. Finally, flies carrying all three groups of amp deletions had no defects in cell proliferation. Error bars represent sd. Group a control brains had 4.8 PH3-positive cells per brain, and injured samples had 13 PH3-positive cells (p-value = 0.003). Group C control samples had 2.4 PH3-positive cells, and injured samples had 10 PH3-positive cells (p-value = 0.002). Group ab control flies had 5.3 PH3-positive cells, and injured samples of the same genotype had 12.7 PH3-positive cells per brain (p-value = 0.002). Group bc control brains had 2.5 PH3-positive cells, and injured brains had 7.3 PH3-positive cells (p-value = 0.04). Group abc control brains had 2.7 PH3-positive cells per brain while injured samples had 13.00 PH3-positive cells (p-value = 0.01). At least three brains were imaged for each condition.

Previous work described in [19] showed that chronic activation of the immune system and expression of AMPs in the brain results in neurodegeneration [19], and 13/14 canonical AMPs are significantly upregulated at 4 hr post-PTBI (Figure 1B). We therefore asked whether the levels of AMP gene expression were chronically increased post-PTBI. We used qRT-PCR to measure mRNA levels of three AMP genes upregulated post-PTBI in the bulk RNA-Seq experiment. We quantified these mRNAs 2, 4, 8, and 24 hr after injury (Figure 3A). Expression levels of *CecB, and DptA*, were increased 10-fold by 4 hr after injury while *Dro* mRNA increases approximately 6-fold by 2 hr after injury. The transcript levels for each of these genes returned towards baseline 24 hr after injury, indicating that the peak of expression is shortly after injury. Taken together, these data show that the immune system is rapidly and acutely activated upon injury and is quickly downregulated. This temporal dynamic could play an important role in inducing proliferation and regeneration as opposed to the neurodegeneration observed with chronic activation of the AMPs. Consistent with the idea that Imd and Toll pathway activation are transient following PTBI, mRNA expression for the Imd pathway transcription factor Rel peaks at 2 hr post-PTBI, while mRNA expression for the Toll pathway ligand Spz peaks at 8 hr post-PTBI (Figure 1C). Transcript levels for both genes also returned to baseline within 24 hr. Their rapid downregulation may be facilitated by inhibition; consistent with this, mRNAs for the negative regulators of the Imd pathway, PGRP-SC2 and Pirk, as well as a negative regulator of the Toll pathway, Spn88Ea are rapidly and transiently upregulated post-PTBI (Figure 1C).

### Canonical targets of Toll and Imd signaling are not cell proliferation effectors

AMPs are a standard readout of innate immune system activation in *Drosophila* and can coordinate cellular responses, including neurodegeneration [19]. If AMPs are required for cell proliferation after injury, we would expect to see a reduction in the number of proliferating cells when AMP expression cannot be stimulated. Fourteen of the 21 AMPs have been classified into three groups [34]. Group A is composed of *Defensin* (*Def*) and the 4 *Cecropins* (*Cec*) and is primarily regulated by the Imd pathway, Group B contains *Drosocin* (*Dro*), the two *Diptericins* (*Dipt*) and the four *Attacins* (*Att*) and is also regulated primarily by the Imd pathway. Group C has *Metchnikowin* (*Mtk*) and *Drosomycin* (*Drs*) and is controlled by the Toll pathway [34]. Working with strains in which combinations of 10 of the AMPs were deleted, we subjected flies to the standard PTBI protocol and assayed for cell proliferation using the anti-PH3 antibody 24 hr after injury. We found that deletions for any of the groups, including the loss of all three groups, continued to show the stereotypical >2-fold increase in PH3-positive cells after injury (Figure 3B). Full descriptions of these stocks are available in Hanson et al. [34]. These results indicate that these 10 AMPs are not the targets of Toll or Imd signalling required for triggering cell proliferation post-PTBI and that there are likely other, non-canonical, targets that play an essential role in stimulating regeneration in adult brains.

We note that the Cecropins were not deleted in the Group A stocks used here and also that the remaining six AMPs were not upregulated after PTBI and therefore not tested. Because these compound mutants did not include the *Cecropin* genes, we cannot rule out a role for Cecropins in activating cell proliferation post-PTBI. However, preliminary experiments with stocks deleted for all 14 AMPs indicate that they also exhibit proliferation following PTBI. Consistent with this, *CecB* expression is upregulated post-PTBI in both *Dif* and *Rel* mutants (not shown), both of which lack a proliferative response after injury (Figure 2). In addition, although the baseline expression of multiple AMPs is reduced in *Dif* and *Rel* mutants, their expression is nonetheless stimulated by injury (not shown) consistent with the hypothesis that non-canonical targets of Dif and Rel activate cell proliferation after PTBI.

### Toll and Imd signaling are required in specific tissues for cell proliferation after injury

**Figure 4.**
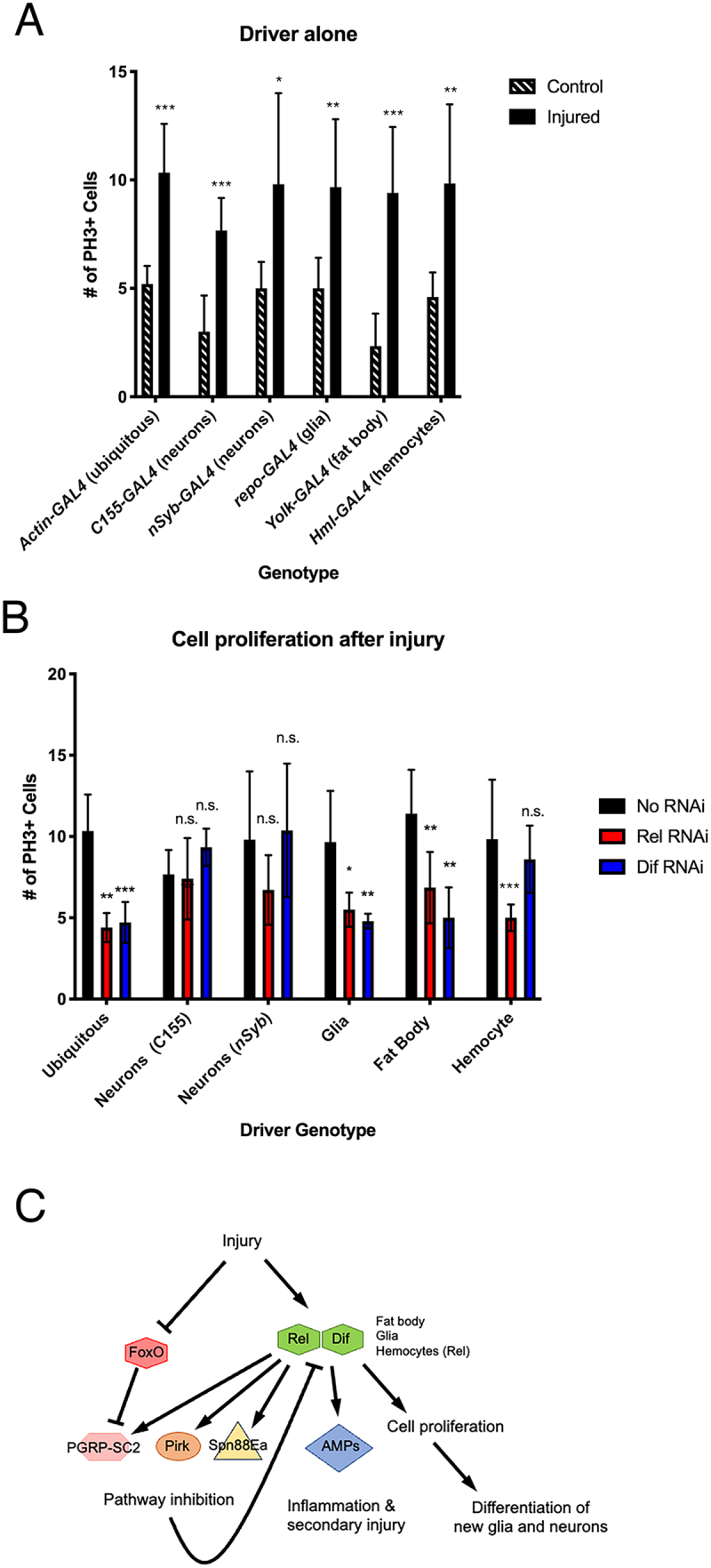
Tissue specific knockdown of *Dif* and *Rel* reveals different requirements for the Toll and Imd pathways in activating cell proliferation following PTBI. A, B. To test where the Toll and Imd innate immunity pathways are required, *Dif* or *Rel* expression were knocked down using UAS-RNAi transgenes. Ubiquitous knockdown was achieved with *Actin-GAL4*, neuronal knockdown with *nSyb-GAL4* and *C155-GAL4*, glial knockdown with *repo-GAL4*, fat body knockdown with *Yolk-GAL4* and hemocyte knockdown with *Hml-GAL4*. Controls for this experiment were Drosophila harboring the GAL4 drivers but lacking RNAi constructs (A). There was a significant increase in the number of proliferating cells after injury in all of the control strains (A). However, there was reduced cell proliferation after injury when *Dif* expression was reduced ubiquitously, in glia, or in the fat body (B). There also was reduced cell proliferation after injury when *Rel* expression was reduced ubiquitously, in glia, or in the fat body (B). In contrast to what was observed with *Dif*, *Rel* knockdown in hemocytes also showed an effect (B). Error bars reflect SD. At least five brains were imaged per condition and genotype, and the numbers of PH3-positive cells are included in Tables S1 and S2. C. Proposed model for the regulation of cell proliferation after PTBI. Injury stimulates the Toll and Imd pathways, resulting in increased expression of *Dif* and *Rel*. Injury leads to downregulation of *FoxO* via an unknown mechanism. Reduction of FoxO is correlated with de-repression of the Imd pathway inhibitor PGRP-SC2 (Guo, Karpac et al. 2014). Dif and Rel activate expression of the AMP genes and the pathway repressors *pirk*, *PGRP-SC2*, and *Spn88Ea*. Pirk and PGRP-SC2 negatively regulate the Imd pathway, while Spn88Ea negatively regulates the Toll pathway, together leading to downregulation of the *AMP* genes by 24 hr post-PTBI. *AMP* downregulation is essential to prevent cell death and neurodegeneration. Injury also stimulates cell proliferation via non-canonical Dif and Rel targets, leading to the generation of new glia and new neurons. We note that FlyBase conventions for the standardization of signaling pathway components (div http://flybase.org/lists/FBgg/pathways div) are followed in the model.

There are multiple tissues present in the *Drosophila* head capsule are impacted by PTBI. Specifically, in addition to the neurons and glia of the brain, two tissues known to be important in the innate immune response are located in the head. These are the fat body, a major site of AMP production following injury and infection [41–43], and haemocytes, which phagocytose debris, and also produce AMPs [44]. To determine the tissue-specific requirements for Toll and Imd signalling to stimulate cell proliferation following injury, we designed tissue-specific knockdown experiments. We utilized the GAL4-UAS binary system [45] to exclusively reduce *Dif* or *Rel* expression via RNA interference (RNAi). We reduced Dif and Rel levels throughout the animal (*Actin-GAL4*), in neurons (*C155-GAL4*), in glia (*Repo-GAL4*), in fat body (*yolk-GAL4*), or in haemocytes (*Hml-GAL4*) and compared the number of proliferating cells per brain after PTBI to controls (Figure 4A,B, quantification in Table S1 and Table S2). The ubiquitous knockdown of either *Dif* or *Rel* is sufficient to reduce the number of proliferating cells after injury compared to injured controls (Figure 4A,B), phenocopying the results we obtained from the *Dif* and *Rel* mutants (Figure 2). Neither *Dif* nor *Rel* knockdown in neurons significantly reduced the amount of cell proliferation after injury (Figure 4B), suggesting that immune signalling in neurons is not required for proliferation after injury. In contrast, immune signalling via both Toll and Imd pathways is required in both glia and fat body, as there was a significant reduction in the number of PH3+ cells when *Dif* or *Rel* were knocked down in either tissue (Figure 4B). Interestingly, *Rel*, but not *Dif*, knockdown in haemocytes was sufficient to prevent cell proliferation after injury (Figure 4B), suggesting that Imd and Toll may be involved in different processes in distinct tissues. Together, these data indicate that the innate immune pathways are required in multiple tissues both within the damaged brain and outside of it to coordinate the activation of cell proliferation that gives rise to new neural tissue.

## Discussion

Neural regeneration after injury is a complex process that likely involves multiple pathways to coordinate cell proliferation, differentiation, and integration of cells into proper circuits. To begin to dissect the mechanisms underlying neural regeneration, we focused on the initial cell proliferative response within 24 hr of injury. We found that there is a rapid and dramatic induction of both the Toll and Imd innate immunity pathways prior to cell division. We therefore asked whether these pathways were required for the neural injury response. Indeed, mutants for either of the NF-κB effectors of the Toll and Imd pathways, *Dif* and *Rel,* respectively, display defects in the induction of cell proliferation after injury, indicating that both Toll and Imd signalling are necessary for the initial injury response. Signalling is required specifically in the fat body and glia for both pathways, with an additional role for Imd activation in haemocytes, indicating requirements for signals from both within the injured brain and outside of it. We also found that the 14 canonical AMPs, the major readouts of immune signalling, are dispensable for activating cell proliferation. We therefore propose that non-canonical downstream targets are responsible for this aspect of Toll and Imd signalling after PTBI.

There is precedence for innate immune signalling in regulating tissue repair after injury.

For instance, injury of the *Drosophila* embryonic epidermis activates Toll signalling, which is required to induce the barrier repair genes [30,31]. In *Drosophila* larvae, Toll signalling is triggered by haemocytes that have been recruited to tumours or damaged regions and coordinates with other pathways involved in survival and death, such as the c-Jun N-terminal kinase (JNK) pathway [46]. Further, this activation of Toll and JNK leads to activation of the Janus kinase/signal transducers and activators of transcription (JAK/STAT) pathway [46,47]. In our 4-hr post-PTBI RNA-Seq data, we observed increased expression of multiple JNK pathway components, including *Matrix metalloproteinase 1* (*Mmp1*) and *Ets21C*. We also observed upregulation of activators of the JAK/STAT pathway, including *unpaired 2* (*upd2*) and *unpaired 3* (*upd3*) as well as other pathway components [48] including *Suppressor of cytokine signaling at 36E*, *regucalcin*, *Nop56* and *noktochor*. Both Rel and STAT92E are required for expression of the pro-survival gene *Turandot A* (*TotA*) [49] which is upregulated ~3-fold after PTBI in our 4-hr bulk RNA-Seq samples. Although the transcription factor Stat92E, a major downstream effector of JAK/STAT, is not upregulated in our 4-hr bulk RNA-Seq samples, it is present. Thus, it is possible that Toll and Imd are working in coordination with JNK and/or JAK/STAT pathways to stimulate a regenerative response in the injured brain. Further, the activation of distinct signalling pathways may depend on activity both in the damaged brain and in other tissues, including the fat body. It will be important to investigate the extent to which these other pathways contribute to the regenerative response, first by determining whether they are required for proliferation after PTBI and then asking whether they are needed in the same or different tissues than Toll and Imd. This could provide insight into why therapies that rely on activation of a single pathway are not sufficient to induce the repair of damaged tissue.

A critical question raised by this work is how transient activation of the Toll and Imd pathways is achieved. Our RNA-Seq data offer a clue. Specifically, the expression of two negative regulators of the Imd pathway and one negative regulator of the Toll pathway are rapidly and significantly elevated after PTBI. These negative regulators are Pirk, PGRP-SC2, and Serpin88Ea [37,50,51]. Pirk acts intracellularly to downregulate Rel activity. PGRP-SC2 is secreted and therefore can downregulate the Imd pathway both cell autonomously and non-cell autonomously, offering the possibility of direct crosstalk among tissues. Serpin88Ea is a serine protease inhibitor that negatively regulates Toll signalling and inhibits the melanization immune response (reviewed in [52]). We hypothesize that these negative regulators play an essential role in the rapid downregulation of the innate immune system following PTBI, thereby preventing cell death and neurodegeneration (Figure 4C) and facilitating neurogenesis. Going forward, this could be tested by reducing or eliminating the activity of these negative regulators and asking whether this prevents cell proliferation post-PTBI. Both Pirk and PGRP-SC2 are known targets of Rel. Thus, activation of the Imd pathway may be self-limiting. Significantly, neither Pirk nor PGRP-SC2 is upregulated following a closed head TBI [23]. One possible explanation for this involves differential regulation of FoxO. FoxO is a known repressor of PGRP-SC2 [53] that is unchanged after a closed head TBI [23], slightly down-regulated (−16%; adjusted P-value = 0.06) in our 4-hr post-PTBI RNA-Seq dataset. Thus, it is possible that the reduction in FoxO levels combines with an increase in Rel activity to activate expression of Imd pathway inhibitors, including PGRP-SC2 (Figure 4C).

mmune activation and inflammation after CNS injuries or the onset of neurodegenerative disorders was long thought to have exclusively negative consequences. Activation of glia is associated with high levels of cytokine production and blood–brain barrier (BBB) permeability in mammals, compromising animal health (reviewed in [54]). In particular, neuroinflammation reduces the proliferation of neural stem cells and decreases the survival and integration of newly created neurons [55]. However, other experiments have uncovered a role for innate immune signalling in promoting repair. Mice that lack IL-6 or TNF, immune signals that are associated with the inflammatory response, have a higher mortality after closed-head injury, indicating that there may be a protective role for immune system activation after TBI [56]. After damage to the olfactory neurons, TNF-α and its downstream effector RelA, a mammalian homolog of the *Drosophila* Relish protein, are required in the injured cells to trigger the proliferation of nearby cells to repair damage [57]. Consistent with this, transient innate immune activation has also been associated with neurogenesis in mammalian systems [58].

Our *Drosophila* PTBI model may help to uncover links between the immune system and neural regeneration that are also present in mammals. This work provides clues as to how the same innate immune pathways can contribute to both regenerative and degenerative phenotypes following neural injury. Further analysis of the mechanisms underlying modulation of the innate immune response following PTBI are likely to reveal how we might manipulate these pathways to shift the balance from negative effects to more beneficial ones [58]. This work demonstrates the first direct links between the induction of neural regeneration and innate immune signalling in *Drosophila* and supports evidence linking immune signalling in mammalian brains to proliferation and regeneration.

## Supplementary Material

Supplemental Material

## Data Availability

Data associated with this paper and not found herein are deposited at NCBI. The GEO accession number for this dataset is: GSE306282 (https://www.ncbi.nlm.nih.gov/geo/)
