# Supplementary material for: Innate immunity pathways activate cell proliferation after penetrating traumatic brain injury in adult Drosophila
Source: Fly (Austin). 2025 Nov 14;19(1):2586357. doi: 10.1080/19336934.2025.2586357 (PMC12622319; doi:10.1080/19336934.2025.2586357)
Supplement: Supplemental Material [file KFLY_A_2586357_SM3752.docx]

**Table S1. Quantification of PH3-positive cells per brain in knockdown experiments.**

| **Genotype** | **Average of PH3-positive cells per control brain** | **Average of PH3-positive cells per injured brain** | **Significance** |
| --- | --- | --- | --- |
| *Actin-GAL4* | 5.2 | 10.3 | Yes (p-value = 0.0099) |
| *Actin>Dif RNAi* | 3.4 | 4.7 | No |
| *Actin>Rel RNAi* | 4.0 | 4.4 | No |
| *Hml-GAL4* | 4.6 | 9.8 | Yes (p-value = 0.0137) |
| *Hml>Dif RNAi* | 3.6 | 8.6 | Yes (p-value = 0.0015) |
| *Hml>Rel RNAi* | 4.3 | 5.0 | No |
| *nSyb-GAL4* | 5.0 | 9.8 | Yes (p-value = 0.039) |
| *nSyb>Dif RNAi* | 3.5 | 10.4 | Yes (p-value = 0.0019) |
| *nSyb>Rel RNAi* | 2.6 | 6.7 | Yes (p-value = 0.0030) |
| *Repo-GAL4* | 5.0 | 9.7 | Yes (p-value = 0.0078) |
| *Repo>Dif RNAi* | 2.6 | 4.8 | Yes (p-value = 0.0039) |
| *Repo>Rel RNAi* | 3.4 | 5.5 | Yes (p-value = 0.0395) |
| *Yolk-GAL4* | 2.3 | 9.4 | Yes (p-value = 0.0007) |
| *Yolk>Dif RNAi* | 3.0 | 5.0 | No |
| *Yolk>Rel RNAi* | 3.7 | 6.9 | Yes (p-value = 0.0016) |
| *C155-GAL4* | 3.0 | 7.7 | Yes (p-value = 0.0005) |
| *C155>Dif RNAi* | 3.8 | 9.3 | Yes (p-value = 0.0008) |
| *C155>Rel RNAi* | 3.3 | 7.4 | Yes (p-value = 0.0196) |

| **Genotype** | | **Average of PH3-positive cells per injured driver brain** | **Average of PH3-positive cells per injured knockdown brain** | **Significance** |
| --- | --- | --- | --- | --- |
| *Actin-GAL4* | *Dif RNAi* | 10.3 | 4.7 | Yes (p-value = 0.0001) |
|  | *Rel RNAi* |  | 4.4 | Yes (p-value = 0.0004) |
| *Hml-GAL4* | *Dif RNAi* | 9.8 | 8.6 | No |
|  | *Rel RNAi* |  | 5.0 | Yes (p-value = 0.0057) |
| *nSyb-GAL4* | *Dif RNAi* | 9.8 | 10.4 | No |
|  | *Rel RNAi* |  | 6.7 | No |
| *Repo-GAL4* | *Dif RNAi* | 9.7 | 4.8 | Yes (p-value = 0.0078) |
|  | *Rel RNAi* |  | 5.5 | Yes (p-value = 0.0116) |
| *Yolk-GAL4* | *Dif RNAi* | 11.4 | 5.0 | Yes (p-value = 0.0024) |
|  | *Rel RNAi* |  | 6.9 | Yes (p-value = 0.0092) |
| *C155-GAL5* | *Dif RNAi* | 7.7 | 9.3 | No |
|  | *Rel RNAi* |  | 7.4 | No |

**Table S2. Quantification and Comparison PH3-positive Cells in Injured *GAL4* Driver and Injured RNAi Knockdown Samples**
